# Metabolizable Protein: 1. Predicting Equations to Estimate Microbial Crude Protein Synthesis in Small Ruminants

**DOI:** 10.3389/fvets.2021.650248

**Published:** 2021-06-10

**Authors:** Stefanie Alvarenga Santos, Gleidson Giordano Pinto de Carvalho, José Augusto Gomes Azevêdo, Diego Zanetti, Edson Mauro Santos, Mara Lucia Albuquerque Pereira, Elzania Sales Pereira, Aureliano José Vieira Pires, Sebastião de Campos Valadares Filho, Izabelle Auxiliadora Molina de Almeida Teixeira, Manuela Silva Libânio Tosto, Laudi Cunha Leite, Lays Débora Silva Mariz

**Affiliations:** ^1^School of Veterinary Medicine and Animal Science, Universidade Federal da Bahia, Salvador, Brazil; ^2^Department of Agricultural and Environmental Sciences, Universidade Estadual de Santa Cruz, Ilhéus, Brazil; ^3^Department of Animal Science, Instituto Federal de Educação, Ciência e Tecnologia do Sul de Minas Gerais, Pouso Alegre, Brazil; ^4^Center of Agrarian Sciences, Universidade Federal da Paraíba, Areia, Brazil; ^5^Department of Plant and Animal Sciences, Universidade Estadual do Sudoeste da Bahia, Itapetinga, Brazil; ^6^Department of Animal Science, Universidade Federal do Ceará, Fortaleza, Brazil; ^7^Department of Animal Science, Universidade Federal de Viçosa, Viçosa, Brazil; ^8^Department of Animal Science, Universidade Estadual Paulista, Jaboticabal, Brazil; ^9^Department of Agricultural and Environmental Sciences, Universidade Federal do Recôncavo da Bahia, Cruz das Almas, Brazil

**Keywords:** bacteria, goat, microorganisms, sheep, rumen, yield

## Abstract

Microbial crude protein (MCP) produced in rumen could be estimated by a variety of protocols of experimental sampling and analysis. However, a model to estimate this value is necessary when protein requirements are calculated for small ruminants. This model could be useful to calculate rumen degradable protein (RDP) requirements from metabolizable protein (MP). Then, our objective was to investigate if there is a difference in MCP efficiency between sheep and goats, and to fit equations to predict ruminal MCP production from dietary energy intake. The database consisted of 19 studies with goats (*n* = 176) and sheep (*n* = 316), and the variables MCP synthesis (g/day), total digestible nutrients (TDN), and organic matter (OM) intakes (g/day), and OM digestibility (g/kg DM) were registered for both species. The database was used for two different purposes, where 70% of the values were sorted to fit equations, and 30% for validation. A meta-analytical procedure was carried out using the MIXED procedure of SAS, specie was considered as the fixed dummy effect, and the intercept and slope nested in the study were considered random effects. No effect of specie was observed for the estimation of MCP from TDN, digestible Organic Matter (dOM), or metabolizable energy (ME) intakes (*P* > 0.05), considering an equation with or without an intercept. Therefore, single models including both species at the same fitting were validated. The following equations MCP (g/day) = 12.7311 + 59.2956 × TDN intake (AIC = 3,004.6); MCP (g/day) = 15.7764 + 62.2612 × dOM intake (AIC = 2,755.1); and MCP (g/day) = 12.7311 + 15.3000 × ME intake (AIC = 3,007.3) presented lower values for the mean square error of prediction (MSEP) and its decomposition, and similar values for the concordance correlation coefficient (CCC) and for the residual mean square error (RMSE) when compared with equations fitted without an intercept. The intercept and slope pooled test was significant for equations without an intercept (*P* < 0.05), indicating that observed and predicted data differed. In contrast, predicted and observed data for complete equations were similar (*P* > 0.05).

## Introduction

Microbial crude protein (MCP) synthesized in the rumen is dependent on the energy supply (1–4). It is known that MCP synthesis is maximized when fermentable carbohydrates and N compounds are supplied synchronously (5, 6). Therefore, main feeding systems preconize models to estimate MCP based on energy compounds as digestible OM (dOM), metabolizable energy (ME), or TDN (7–13), once these models could be used to calculate rumen degradable protein (RDP) requirements from metabolizable protein (MP) requirements. Moreover, the general importance of the estimation of microbial protein for ruminants must be considered, as it accounts for between 50 and 90% of the protein entering the duodenum and provides most of the amino acids needed for growth and milk protein synthesis (14, 15).

According to Dijkstra et al. (16), empirical equations in most protein evaluation systems usually consider MCP synthesis calculated from the amount of available energy, applying a fixed or variable yield of microbial protein formed per unit energy degraded. As reported by Galyean and Tedeschi (4), the validity of predicting MCP synthesis from TDN intake using the factor of 0.13, mostly used in American systems for cattle, has been neither extensively assessed experimentally nor evaluated. Hence, theses authors proposed equations with an intercept included. On the other hand, this information for goats and sheep is scarce, and little information could be found in the literature. NRC (9) proposed to predict crude protein (CP) requirements without an equation to estimate MCP and RDP requirements, which was based on percentage true protein in MCP, in average CP digestibility, and the percentage of undegradable dietary protein (UDP) in CP. This kind of simplification could reduce the accuracy of nutrient requirement predictions in sheep and goats, mainly when using tropical forages. Diets based on low-quality forages usually require an improved synchronization between energy and protein to achieve the maximum degradation profile.

When sheep and goats are compared in the fermentation process without diet interference, little differences are observed in pH, volatile fatty acids, and ammonia nitrogen concentrations in rumen fluid. However, interspecies differences in ruminal microbial composition are found, favoring goats in the concentration of rumen microbial protein (17, 18). Carro et al. (18) compared methods to estimate MCP in sheep and goats and reported that there were no interspecies differences in total purine derivative excretion in urine. Besides, no differences were detected when ruminal bacteria production (liquid or solid associated) was compared between species, and no dietary interactions were found in this research. Then, our objective was to investigate if there is a difference in MCP efficiency between sheep and goats, and to fit equations to predict ruminal MCP production from dietary energy intake.

## Materials and Methods

### Database Description

**Figure 1:**
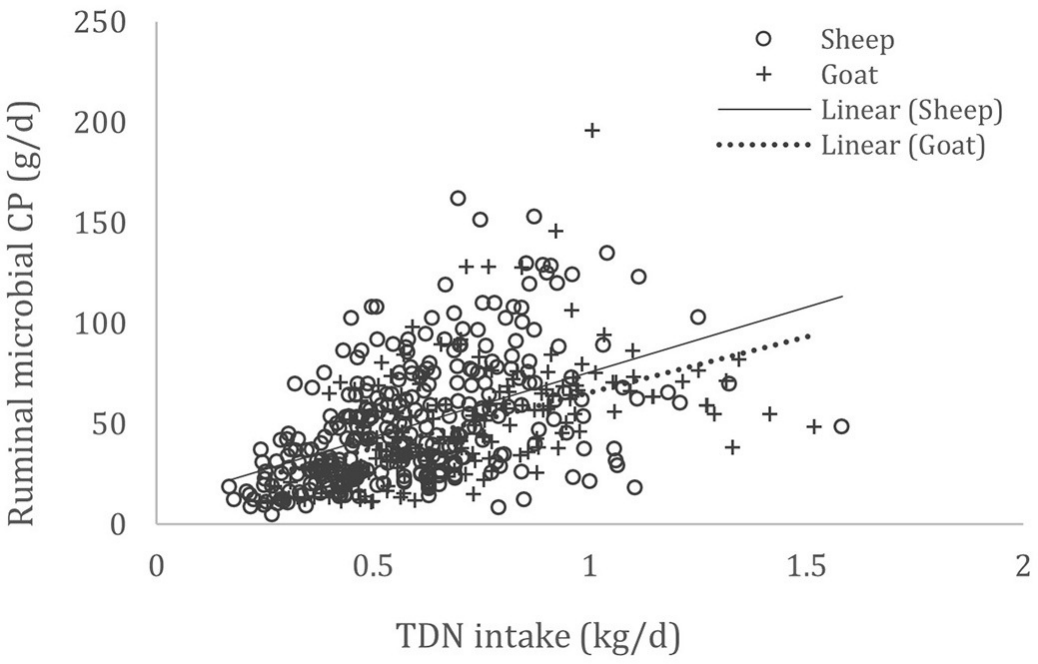
Total databank used to establish the relationship between total digestible nutrient (TDN) intake (kg/day) and microbial crude protein (MCP—g/day) produced in the rumen of sheep and goats, made from 19 experiments (*n* = 492).

**Figure 2:**
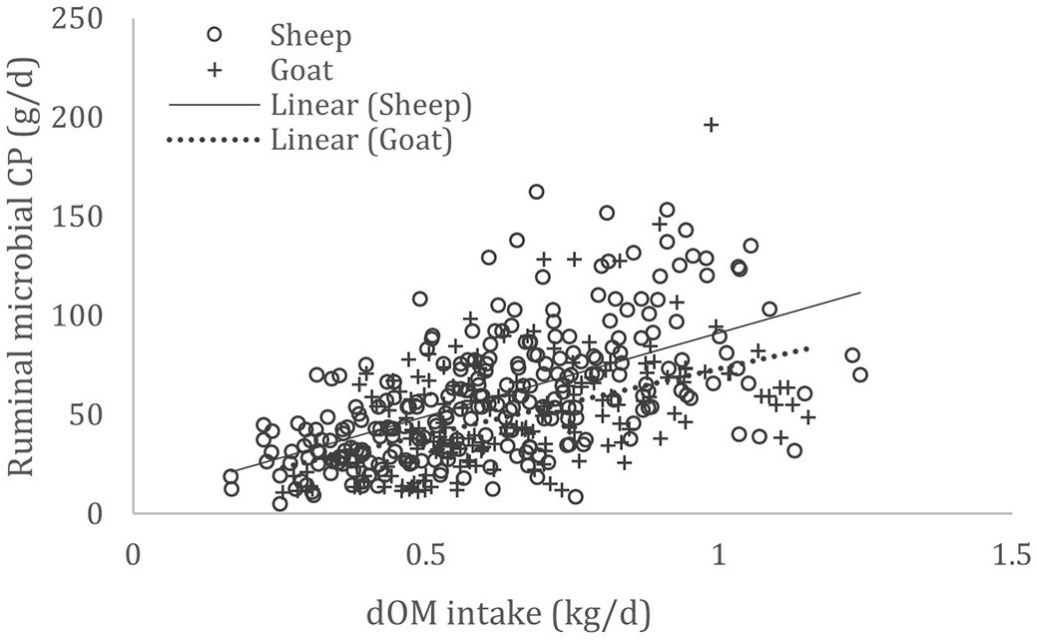
Total databank used to establish the relationship between digestible organic matter (dOM) intake (kg/day) and microbial crude protein (MCP—g/day) produced in the rumen of sheep and goats, made from 18 experiments (*n* = 435).

**Figure 3:**
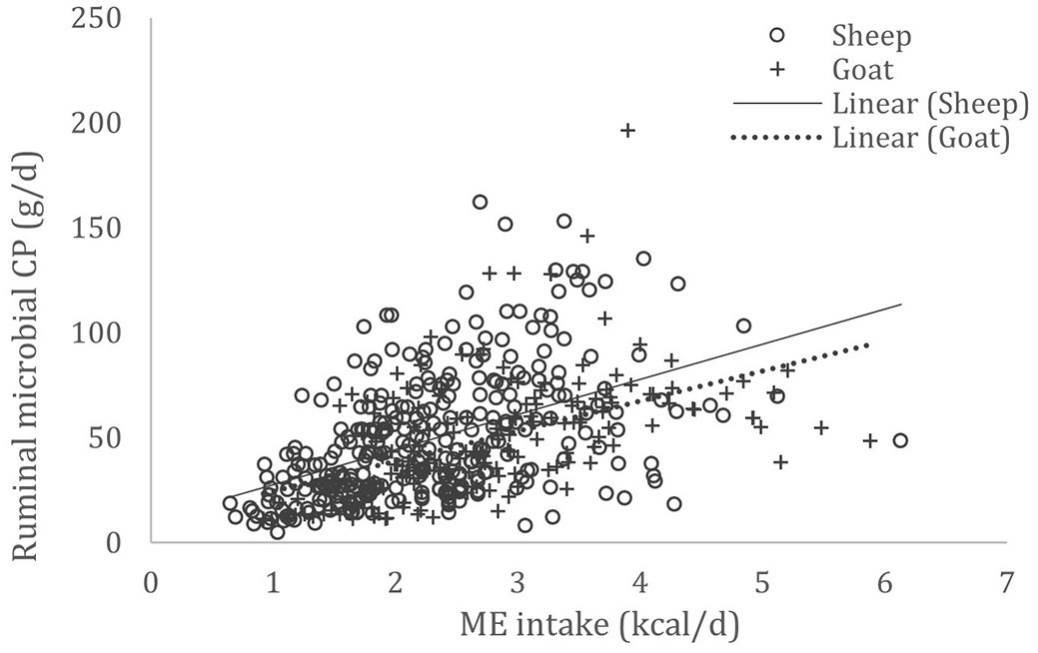
Total databank used to establish the relationship between metabolizable energy (ME) intake (Mcal/day) and microbial crude protein (MCP—g/day) produced in the rumen of sheep and goats, made from 19 experiments (*n* = 492).

A complete database composed of individual experimental units from sheep and goat trials was obtained to conduct a meta-analysis. The spreadsheet was composed of general information (e.g., title, author name, breed, sex, treatment, type of diet) and all necessary quantitative data. The original database consisted of 19 studies (Appendix) with goats (6 studies), sheep (12 studies), and with both species (1 study), conducted at the Federal University of Bahia, Bahia Southwest State University, Federal University of Ceará, Federal University of Paraiba, and Santa Cruz State University facilities between 2010 and 2019. The detailed descriptive statistics of the database filtered for each nutrient evaluation were described in Table 1. Data were collected when MCP synthesis (g/day), TDN intake (kg/day) (Figure 1), OM intake (kg/day) (Figure 2), CP intake (kg/day), and OM digestibility (g/kg DM) were measured for sheep or goat. The TDN data were converted in ME (Figure 3) by the constants 4.404 (8), which converts TDN to digestible energy (DE), and 0.88 (19), which converts DE in ME, resulting in the following conversion factor: ME intake (Mcal/day) = 3.87552 × TDN intake (kg/day).

**Table 1:** Descriptive statistics of the variables used to fit and validate models to estimate microbial crude protein efficiency in the rumen of goat and sheep species from energy intake measurements.

Item^*^	**Goat**					**Sheep**					**Total**	
	**Mean**	**Minimum**	**Maximum**	***SD***	***N***	**Mean**	**Minimum**	**Maximum**	***SD***	***N***	**Subject**	***N***
**TOTAL DATA**												
MCP	49.59	10.75	196.25	27.81	176	51.13	4.98	162.26	32.35	316	19	492
TDN intake	0.71	0.24	1.51	0.25	176	0.59	0.17	1.58	0.22	316	19	492
dOM intake	0.64	0.25	1.15	0.20	176	0.61	0.17	1.24	0.22	259	18	435
ME intake	2.75	0.92	5.87	0.98	176	2.29	0.65	6.13	0.87	316	19	492
**FITTING EQUATIONS**												
MCP	49.45	10.74	196.25	28.63	123	53.71	8.27	153.12	31.64	216	19	339
TDN intake	0.70	0.25	1.41	0.25	123	0.61	0.18	1.58	0.23	216	19	339
dOM intake	0.63	0.25	1.13	0.20	123	0.64	0.16	1.24	0.21	180	18	303
ME intake	2.70	0.99	5.48	0.99	123	2.37	0.69	6.13	0.88	216	19	339
**VALIDATING EQUATIONS**												
MCP	49.92	11.47	146.07	26.05	53	44.63	4.98	162.27	30.52	100	19	153
TDN intake	0.74	0.24	1.52	0.24	53	0.54	0.17	1.18	0.21	100	19	153
dOM intake	0.67	0.27	1.15	0.19	53	0.54	0.17	1.13	0.23	79	18	132
ME intake	2.87	0.92	5.88	0.87	53	2.11	0.65	4.57	0.82	100	19	153

* *MCP, microbial crude protein synthesized in rumen (g/day); TDN, total digestible nutrients; dOM, digestible organic matter; ME, metabolizable energy*.

### Animals and Feeding Management of the Digestibility Trials

The animals included in the database were some of the most common genotypes of sheep and goat species used in tropical systems: pure breed Santa Inês sheep, crossbred sheep (Santa Inês × Dorper; Santa Inês × local non-defined breed), Morada Nova sheep, Bergamacia sheep, Boer goats, crossbred Boer goats × dairy breeds, and Saanen and Anglo-Nubian goats. In all experiments evaluated, animals were managed under tropical conditions, in feedlot management, and fed *ad libitum*.

The forage sources included Tifton-85 hay, Buffel grass hay, Cassava hay, Elephant grass silage, sorghum silage, corn silage, and pearl millet silage. The concentrate components included soybean hulls, peanut cake, cottonseed cake, palm kernel cake, detoxified castor cake, spineless cactus, cottonseed hulls, ground corn, soybean meal, and crude glycerin. All animals were supplemented with a mineral mixture in the concentrate components. The relationship between forage and concentrate ranged from 200 to 700 g of forage/kg of fresh feed. Nutrient intake was recorded daily in all the experiments, and values per animal for each experiment were recorded into the database. At least organic matter (OM) digestibility or total digestible nutrients (TDN) were required to incorporate the study in the database. Digestibility trials that generate these data were carried out using total feces collection or its prediction using different internal markers. Microbial crude protein production was measured by the purine derivative method or rumen bacterial RNA isolation.

### Calculations and Statistical Methods

The database was divided into two subsets, in which 70% of the data were used to fit the proposed models, and 30% were used to validate the equations. Data were randomly divided using the Excel software, where both subsets had data from all 30 experiments.

Two different approaches were used to predict MCP production, where independent variables were the TDN intake and the dOM intake. The TDN intake was used to calculate the ME intake, generating a third approach. These constructions were made based on 70% of the dataset separated for this finality. All models were evaluated with and without an intercept. The effect of animal specie (sheep or goat) was investigated by adding a dummy variable (20). A complete model was defined to consider also the inter-experiment variation, as follows:

(1) $$\begin{array}{l} Y_{ijk}=\alpha_{1}+\alpha_{2}D_{i}+\beta_{1}X_{ijk}+\beta_{2}X_{ijk}D_{i}+E_{\left(i\right)j}+\;\varepsilon_{ijk} \end{array}$$

where *Y*_*ijk*_ = amount of MCP in the rumen (g/day) in the *k*^th^ observation into experiment *j*^th^ and specie *i*^th^; *X*_*ijk*_ = Intake of TDN, dOM, or ME (kg/day) in the *k*^th^ observation into experiment *j*^th^ and specie *i*^th^; *Di* = dummy variable referent to the *i*^th^ animal species with *D* = 0 for sheep and *D* = 1 for goat; ß_1_ and ß_2_ = estimated slopes for sheep and goats, respectively; α_1_ and α_2_ = estimated intercepts for sheep and goats, respectively; *E*_(*i*)*j*_ = effect of the *j*^th^ experiment nested with the *i*^th^ animal specie; and ε_*ijk*_ = random error.

In case the parameters associated with the dummy variables would not be statistically significant for some independent variables tested, a reduced model was fitted without considering the *D* effect, as follows:

(2) $$\begin{array}{l} Y_{ijk}=\alpha_{1}+\beta_{1}X_{ijk}+E_{\left(i\right)j}+\;\varepsilon_{ijk} \end{array}$$

where *Y*_*ijk*_ = amount of MCP in the rumen (g/day) in the *k*^th^ observation into experiment *j*^th^ and specie *i*^th^; *X*_*ijk*_ = intake of TDN, dOM, or ME (kg/day) in the *k*^th^ observation into experiment *j*^th^ and specie *i*^th^; β_1_ = estimated slope for small ruminants; α_1_ = estimated intercept for small ruminants; *E*_(*i*)*j*_ = effect of the *j*^th^ experiment nested with the *i*^th^ animal specie; and ε_*ijk*_ is the random error.

Same inferences were produced to evaluate the above models without the intercept. Theoretically, the absence of an intercept could make biological sense because, in the absence of energy intake (slope = 0), represented by TDN, dOM, and ME, no amount of MCP could be produced, and then two other approaches were tested, as follows:

(3) $$\begin{array}{l} Y_{ijk}=\beta_{1}X_{ijk}+\beta_{2}X_{ijk}D_{i}+E_{\left(i\right)j}+\;\varepsilon_{ijk} \end{array}$$

(4) $$\begin{array}{l} Y_{ijk}=\beta_{1}X_{ijk}+E_{\left(i\right)j}+\;\varepsilon_{ijk} \end{array}$$

Meta-analysis techniques (21) were applied. The MIXED procedure of SAS 9.2 (22) was used to estimate the coefficients, considering the influence of the species on the intercept and the slope (*D* = 0 for sheep and *D* = 1 for goat). The random effects of the experiments were considered both on the intercept and slopes (SUBJECT = study) according to Huhtanen et al. (23). It should be emphasized that linear regression models were analyzed considering an unstructured (co)variance matrix (TYPE = UN). All variance components in the linear models were estimated using the restricted maximum likelihood method. For all statistical procedures, the critical level of the probability for type-I error was fixed at 0.05.

### Validation Process

Evaluation of the equations was carried out from the 30% remaining subset. A total of 153 experimental units were used in this process, and the completely descriptive characterization of these data was presented in Table 1. Mathematical comparisons were performed using the MES software (Model Evaluation System, version 3.1, Texas A&M University, College Station, TX, USA) according to Tedeschi (24), after calculating Mayer's intercept and slope test (25), the root mean square of error (RMSE), the concordance correlation coefficient (CCC), the mean bias, slope bias, and random errors decomposed from the mean square error of prediction (MSEP), the predicted mean, and the mean deviation. All statistical procedures were performed using 0.05 as the critical level of probability for type I error occurrence.

## Results

A complete model with an intercept was adjusted to estimate MCP production (g/day), considering TDN intake (kg/day) as the independent variable (Table 2). The inclusion of both a slope (*P* = 0.39) and an intercept (*P* = 0.88) associated with the dummy variable did not improve the equation (AIC = 2,989.7). The specie factor was not statistically necessary to construct an adequate model. Therefore, the reduced model presentation (AIC = 3,004.6) had both a slope and an intercept fitted (*P* < 0.01), producing the following equation:

(5) $$\begin{array}{l} \text{MCP }(\frac{\text{g}}{\text{day}})=12.7311_{5.2307}+59.2956_{9.1130}\;\times \text{ TDNI }\left(\frac{\text{kg}}{\text{day}}\right) \end{array}$$

The same scenario was observed when the TDN intake model was fitted without an intercept (Table 2). The inclusion of the slope (*P* = 0.23) associated with the dummy variable did not improve the equation (AIC = 3,012.9). The specie factor was also not statistically necessary to construct an adequate model. Therefore, the reduced model presentation (AIC = 3,021.5) had the slope fitted (*P* < 0.01) and can also be considered as an alternative equation:

(6) $$\begin{array}{l} \text{MCP }\left(\frac{\text{g}}{\text{day}}\right)=78.2875_{7.0036}\;\times \text{ TDNI }\left(\frac{\text{kg}}{\text{day}}\right) \end{array}$$

The two above equations (Equations 5 and 6) were evaluated by a validation process (Table 3). Predicted and observed data from the equation fitted with an intercept (Equation 5) for TDN intake were similar (*P* = 0.43). Therefore, it is possible to infer that this equation is adequate to predict MCP in sheep and goats. The same inference could not be made for the equation (Equation 6) without an intercept (Table 3), which had a difference between predicted and observed data (*P* = 0.04). Comparison relative to MSEP and its decomposition was also positive for the equation with an intercept (Equation 5), once MSEP was lower for that, and only 0.06% slope bias was counted. Besides, random errors prevailed in the equation with an intercept for TDN intake (Equation 5), which is suitable. However, both equations (Equations 5 and 6) with or without an intercept had no difference in the predicted mean (*P* > 0.05) and similar values for CCC and RMSE.

**Table 2:** Estimated parameters and statistic evaluation of the relationships between intake of total digestible nutrients (TDN), of digestible organic matter (dOM), and of metabolizable energy (ME) intakes with ruminal microbial crude protein in complete (different parameters for each specie) and reduced (same parameters for both species) modes, where the letter “D” represents a dummy variable (D = 0 for goat and D = 1 for sheep), with models with and without intercept (Noint).

**Effect**	**Estimate**	**SEM**	***P*-value**	**AIC**
**TOTAL DIGESTIBLE NUTRIENTS INTAKE**				
**Complete**				
Intercept	13.5377	9.0311	0.15	2,989.7
D * intercept	−1.6168	11.2017	0.88	
Slope	49.8173	14.6926	<0.01	
D * slope	15.9174	18.8767	0.39	
**Reduced**				
Intercept	12.7311	5.2307	0.03	3,004.6
Slope	59.2956	9.1130	<0.01	
**Complete Noint**				
Slope	67.5505	11.3826	<0.01	3,012.9
D * slope	17.0811	14.3600	0.23	
**Reduced Noint**				
Slope	78.2875	7.0036	<0.01	3,021.5
**DIGESTIBLE ORGANIC MATTER INTAKE**				
**Complete**				
Intercept	16.5212	9.5453	0.10	2,739.7
D * intercept	−1.1159	12.3614	0.93	
Slope	51.8497	14.4974	<0.01	
D * slope	16.7819	18.5136	0.36	
**Reduced**				
Intercept	15.7764	6.0458	0.02	2,755.1
Slope	62.2612	9.1100	<0.01	
**Complete Noint**				
Slope	76.2621	8.3169	<0.01	2,761.4
D * slope	14.9254	10.6158	0.16	
**Reduced Noint**				
Slope	85.4081	5.3266	<0.01	2,769.9
**METABOLIZABLE ENERGY INTAKE**				
**Complete**				
Intercept	13.5377	9.0311	0.15	2,995.2
D * intercept	−1.6168	11.2017	0.88	
Slope	12.8544	3.7911	<0.01	
D * Slope	4.1072	4.8707	0.39	
**Reduced**				
Intercept	12.7311	5.2307	0.03	3,007.3
Slope	15.3000	2.3514	<0.01	
**Complete Noint**				
Slope	17.4301	2.9371	<0.01	3,018.3
D * slope	4.4074	3.7053	0.23	
**Reduced Noint**				
Slope	20.2005	1.8071	<0.01	3,024.2

**Table 3:** General evaluation of the proposed models, with or without intercept (Noint), to estimate microbial crude protein efficiency in sheep and goat species from energy intake measurements.

**Equation**	**TDN intake**	**dOM intake**	**ME intake**	**TDN intake Noint**	**dOM intake Noint**	**ME intake Noint**
Mayer's test						
Intercept	−0.44	−9.05	−0.44	11.72	9.07	11.72
Slope	0.95	1.15	0.95	0.72	0.84	0.72
*P*-value	0.43	0.57	0.43	0.04	0.36	0.04
RMSE	25.62	26.21	25.62	26.14	26.32	26.14
CCC	0.37	0.40	0.37	0.43	0.48	0.43
MSEP	658.95	683.45	658.95	681.48	688.23	681.48
Mean bias %	1.05	0.20	1.05	0.35	0.10	0.35
Slope bias %	0.06	0.65	0.06	4.03	1.45	4.03
Random errors %	98.88	99.14	98.88	95.61	98.45	95.61
Predicted mean	49.10	52.84	49.10	48.02	50.84	48.02
Mean deviation	2.63	1.18	2.63	1.55	−0.81	1.55
*P*-value	0.20	0.60	0.20	0.46	0.72	0.46

*TDN, total digestible nutrients; dOM, digestible organic matter; ME, metabolizable energy; Mayer's test considered the null hypothesis as H0: intercept = 0 and slope = 1, and H1: reject H0; RMSE, Root mean square error; CCC, concordance correlation coefficient; MSEP, mean square error of prediction.

A complete model with an intercept was adjusted to estimate MCP production (g/day), considering dOM intake (kg/day) as the independent variable (Table 2). The inclusion of both a slope (*P* = 0.36) and an intercept (*P* = 0.93) associated to the dummy variable did not improve the equation (AIC = 2,739.7). Therefore, the reduced model presentation (AIC = 2,755.1) had both a slope and an intercept fitted (*P* < 0.05), producing the following equation:

(7) $$\begin{array}{l} \text{MCP }\left(\frac{\text{g}}{\text{day}}\right)=15.7764_{6.0458}+62.2612_{9.1100}\;\times \text{ dMOI }\left(\frac{\text{kg}}{\text{day}}\right) \end{array}$$

The same scenario was observed when the dMO intake model was fitted without an intercept (Table 2). The inclusion of a slope (*P* = 0.16) associated to the dummy variable did not improve the equation (AIC = 2,761.4). Therefore, the reduced model presentation (AIC = 2,769.9) had the slope fitted (*P* < 0.01) and can also be considered as an alternative equation:

(8) $$\begin{array}{l} \text{MCP }\left(\frac{\text{g}}{\text{day}}\right)=85.4081_{5.3266}\;\times \text{ dMOI }\left(\frac{\text{kg}}{\text{day}}\right) \end{array}$$

The two above equations (Equations 5 and 6) were compared by a validation process (Table 3). Predicted and observed data from the equation fitted with an intercept (Equation 7) for dMO intake were similar (*P* = 0.57), which could lead to infer that this equation is adequate to predict MCP in sheep and goats. The same inference could be made for the equation (Equation 8) without an intercept (Table 3), which had no difference between predicted and observed data (*P* < 0.36). Comparison relative to MSEP and its decomposition was positive for the equation with an intercept (Equation 7), once MSEP was lower for that, and a 0.65% slope bias was counted contrasting with 1.45% for the equation without an intercept. Also, random errors prevailed in the equation with an intercept for dMO intake (Equation 7), which is suitable. However, both equations (Equations 7 and 8) with or without an intercept had no difference in the predicted mean (*P* > 0.05) and similar values for CCC and RMSE.

A complete model with an intercept was adjusted to estimate MCP production (g/day), considering ME intake (Mcal/day) as the independent variable (Table 2). The inclusion of both a slope (*P* = 0.39) and an intercept (*P* = 088) associated with the dummy variable did not improve the equation (AIC = 2,995.2). Therefore, the reduced model presentation (AIC = 3,007.3) had both a slope and an intercept fitted (*P* < 0.05), producing the equation

(9) $$\begin{array}{l} \text{MCP }\left(\frac{\text{g}}{\text{day}}\right)=15.3000_{2.3514}+12.7311_{5.2307}\;\times \text{ MEI }\left(\frac{\text{Mcal}}{\text{day}}\right) \end{array}$$

The same scenario was observed when the ME intake model was fitted without an intercept (Table 2). The inclusion of a slope (*P* = 0.23) associated with the dummy variable did not improve the equation (AIC = 3,018.3). Therefore, the reduced model presentation (AIC = 3,024.2) had the slope fitted (*P* < 0.01) and can also be considered as an alternative equation:

(10) $$\begin{array}{l} \text{MCP }\left(\frac{\text{g}}{\text{day}}\right)=20.2005_{1.8071}\;\times \text{ MEI }\left(\frac{\text{Mcal}}{\text{day}}\right) \end{array}$$

The two above equations (Equations 9 and 10) were compared by a validation process (Table 3). Predicted and observed data from the equation fitted with an intercept (Equation 9) for ME intake were similar (*P* = 0.43). Therefore, it is possible to infer that this equation is adequate to predict MCP in sheep and goats. The same inference could not be made for the equation (Equation 10) without an intercept (Table 3), which had a difference between predicted and observed data (*P* = 0.04). Comparison relative to MSEP and its decomposition was also positive for the equation with an intercept (Equation 9), once MSEP was lower for that, and only 0.06% slope bias was counted. Besides, random errors prevailed in the equation with an intercept for MEI (Equation 9), which is suitable. However, both equations (Equations 9 and 10) with or without an intercept had no difference in the predicted mean (*P* > 0.05) and similar values for CCC and RMSE.

## Discussion

The relationship between MCP synthesis and energy available in the rumen was well-documented. The synchronism between protein and energy degradation in the rumen is one of the factors that impacts MCP efficiency (26–30). According to Schwab and Broderick (31), when there is excessive N relative to energy compounds in the rumen, the ruminal N waste increases. This result means that lower levels of N compounds were incorporated to MCP synthesized in the rumen due to the reduced carbohydrate sources. Then, it is known that MCP synthesis is driven by the ruminant's energy intake.

Energy intake and its availability in rumen could be expressed in different forms. The dOM intake directly expresses the energy that can be used by the microbial population in the rumen, once it represents the sum of all nutrients that can generate energy and that can be accessed by microorganisms. The mathematical relationship between MCP synthesis and intake of dOM was established by Gomes et al. (32) when they supplied increasing levels of soluble starch to sheep and observed the increase in dMO intake and microbial N synthesis linearly.

Sinclair et al. (33) obtained similar results in sheep, but these authors highlighted the importance of synchronization between these nutrients to improve the dMO utilization by rumen microorganisms. Stern and Hoover (34) made a compilation of 64 manuscripts published from 1970 to 1979, and they observed a mean value of 16.9 g (±6.2) of MCP synthesized in rumen per 100 g of dOM for sheep and cattle, being the values ranging from 6.3 to 30.7 g of MCP. The authors attributed this difference to the variety of microbial markers used, content and source of N and carbohydrates, rumen dilution rate, dietary sulfur, and feed frequency. In this study, it can be noted through Equation (8) that the relationship found for sheep and goats was 8.26 g of MCP synthesized in rumen per 100 g of dOM, where diversity of dietary types was included in the dataset.

The TDN intake as a base for MCP estimation started to be further investigated also in the 1970s. However, most studies were started in cattle to predict RDP and UDP requirements better. Burroughs et al. (35) proposed that MCP synthesis averaged 13.05% of TDN for cattle. Burroughs et al. (36) reported that estimation of MCP synthesis could be from 10.4% of TDN based upon three evaluations: the first is that approximately 52% of TDN is digested in the rumen; the second is that 25% of the digested TDN becomes MCP when adequate N is present; the third is that 80% of MCP is alpha-amino protein.

According to Hackmann and Firkins (37), the knowledge about energy metabolism in rumen microorganisms is the key to understand how to improve growth efficiency and how to predict microbial protein production. The MCP synthesis prediction is a necessary step to improve the protein requirement' systems. In years later, TDN was adopted as a pattern to predict MCP synthesis and RDP by NRC (38), allowing this system to convert MP requirements into crude protein requirements for beef cattle. This committee refused the two equations proposed by the “Ruminant Nitrogen Usage” committee in 1985 (39), which were based in estimates generated by different forage to concentrate ratios and a negative intercept for the relationship between MCP and TDN [MCP (g/day) = 6.25 – 31.86 + 26.12 × TDN (kg/day)]. NRC (40) highlighted that a negative intercept had no biological logic and that equations based on the forage-to-concentrate ratio are misleading because it suggests that both intake and concentration of TDN change in a similar direction. For dairy cattle, NRC (8) also adopted the constant 130 g MCP/kg TDN for heifers and cows, and they proposed that the RDP requirement could be calculated by 1.18 × MCP yield.

Russell et al. (41), when proposing the CNCPS system, made some criticism concerning TDN for MCP prediction. The authors stated that ruminal microbial growth is driven by available carbohydrates rather than TDN, considering that TDN is also composed of EE and CP besides total carbohydrates, and they would not be the primary source of energy for microorganisms in the rumen. However, according to Lucas and Smart (42), EE and CP and NFC are nutritional entities with constant digestion rate, represented by a slope between consumed and digested nutrients, and with an intercept that represents the endogenous fraction. In other words, NDF is the key nutrient impacting the most variability in TDN, while the other components would be mostly driven by nutrient intake. Thus, it can be seen that the main criticism that 40 directed to TDN could be questioned once TDN can reflect the oscillations in the carbohydrate fermentation rate.

Therefore, even though TDN was questioned for the MCP estimate, the most recent American System for beef cattle (12) continued using the TDN as the MCP predictor, adopting two equations proposed by Galyean and Tedeschi (4). These authors proposed an equation to estimate MCP based only on TDN intake (MCP = 0.087 × TDNI + 42.73) but with an adjustment in the equation parameters when diets have high EE (MCP = 0.096 × TDNI + 53.33). NASEM (12) recognized that microbial growth is measured with high variability, which impacts directly in the standard MSEPs and indirectly in the MP requirement prediction. However, the authors also recognized that this high variability is caused by the available techniques and depends on many other variables that cannot be accounted for by a single empirical equation.

The Brazilian system for beef cattle (11) was based exclusively on data from tropical forages, as well as this work that was made for sheep and goats. The BR-Corte (11) preconized equations based both in TDN and dMO, differing in their quadratic parameters, and CP as a component of those equations. However, it must be emphasized that the data of this work did not fit in this type of model when tested. This lack of fit probably is due to the difference in the specie type, and the number of observations; once the cited system has used 2,102 individual observations, while this work was proposed with 592 observations to fit equations.

For sheep and goats, NRC (9) adopted only one approach. This system presented a basic simplification proposed by Sahlu et al. (43) and imported from NRC (40). NRC (9) stated that even though MP requirements are preferred compared with CP, for some users, the application of MP requirements might be difficult. In the absence of an equation capable to predict MCP for sheep and goats, this system took into consideration that MCP is 0.64 of CP, which corresponds to 80% of true protein and 80% of digestibility, and that 16% of CP is UDP. These premises led to the following simplification: CP = MP/[64 + (0.16 × %UDP)/100], which allowed NRC (9) to predict CP requirements without proposing an equation to estimate MCP and RDP requirements. This kind of simplification could reduce the accuracy of nutrient requirement predictions in sheep and goats, mainly when using tropical forages, which require an improved synchronization between energy and protein to achieve the maximum degradation profile.

CSIRO (10), working with cattle and sheep, proposed a specific equation based on ME intake, applying an adjustment for the intake level above maintenance. This system proposed some adjustments for the latitude of the region, the day of the year, and the type of forage. All the proposed models were exponential, always considering the intake level as an exponential parameter and the ME intake as a slope parameter. In this work, an equation based on the ME intake was also proposed because this is the preferred energy unit for some nutrient requirement systems. The contribution of the adequate MCP prediction is basically to improve dietary balance for sheep and cattle, minimizing environmental N excretions and maximizing animal performance. These findings can contribute to environmental and economic sustainability in small ruminant production systems.

## Conclusions

There are no differences to fit MCP efficiency between sheep and goats. The MCP synthesis in the rumen can be predicted from energy intake using combined equations that encompass both sheep and goats, once the specie was not a factor affecting this prediction. The approaches for this prediction can be based on NDT, dOM, or ME intake by the following equations: MCP (g/day) = 12.7311 + 59.2956 × TDN intake (AIC = 3,004.6); MCP (g/day) = 15.7764 + 62.2612 × dOM intake (AIC = 2,755.1); and MCP (g/day) = 12.7311 + 15.3000 × ME intake (AIC = 3,007.3). The same approaches were tested after deleting intercept parameters from the models, but after the validation process, these equations were not accurate satisfactorily.

## Data Availability Statement

The raw data supporting the conclusions of this article will be made available by the authors, without undue reservation.

## Author Contributions

SS: writing—original draft, data curation, conceptualization, methodology, formal analysis, supervision, and project administration. GC: conceptualization, data curation, and investigation. JA, ES, MP, EP, AP, and LL: data curation and investigation. DZ: validation, methodology, and formal analysis. SV, IT: conceptualization, visualization, and validation. MT and LM: visualization and validation.

## Conflict of Interest

The authors declare that the research was conducted in the absence of any commercial or financial relationships that could be construed as a potential conflict of interest.

## Abbreviations

DM: dry matter
CP: crude protein
RDP: rumen degradable protein
UDP: undegradable dietary protein
MP: metabolizable protein
MCP: microbial crude protein
OM: organic matter
dMO: digestible organic matter
TDN: total digestible nutrients
ME: metabolizable energy
DE: digestible energy
RMSE: root mean square of error
CCC: concordance correlation coefficient
MSEP: mean square error of prediction.
