# Supplementary material for: Metabolizable Protein: 1. Predicting Equations to Estimate Microbial Crude Protein Synthesis in Small Ruminants
Source: Front Vet Sci. 2021 Jun 10;8:650248. doi: 10.3389/fvets.2021.650248 (PMC8222605; doi:10.3389/fvets.2021.650248)
Supplement: Supplementary file 1 [file Table_1.docx]

**APPENDIX**

- Araujo, M. L. G. M. L., de Carvalho, G. G. P., Ayres, M. C. C., Bezerra, L. S., Rebouças, R. A., da Costa Vieira Filho, C. H., Oliveira, R. L., and Costa Teixeira, C. S. (2014). Assessment of the metabolic, protein, energy, and liver profiles of lambs finished in a feedlot and receiving diets containing groundnut cake. Trop. Anim. Health. Prod. 46, 433-437. doi: 10.1007/s11250-013-0510-4

Barbosa, J. S. R., Souza, J. G., Herbster, C. J. L., Silva, L. P., Carvalho J. D. G., Medeiros, A. N., Marcondes, M.I., Bezerra, L. R., Oliveira, R. L., Alves, S. P. Bessa, R. J. B., Pereiraa, E. S. (2021). Basal diets with different starch contents do not modify the metabolism of ricinoleic acid in dairy goats. Anim. Feed Sci. Technol. 276, 1-10. doi: 10.1016/j.anifeedsci.2021.114900

Carvalho, G. G. P. D., Garcia, R., Pires, A. J. V., Silva, R. R., Pereira, M. L. A., Viana, P. T., and Pereira, T. C. D. J. (2010). Nitrogen balance, urea concentrations and microbial protein synthesis in goats fed diets containing sugar cane treated with calcium oxide. Rev. Bras. Zootec. 39, 2253-2261. doi: 10.1590/S1516-35982010001000022

Dos Santos, A. B., Pereira, M. L. A., de Oliveira Silva, H. G., de Carvalho, G. G. P., Ribeiro, L. S. O., de Jesus Pereira, T. C., and Almeida, P. J. P. (2016). Nitrogen metabolism in lambs fed diets containing peach palm meal. Trop. Anim. Health. Prod. 48, 1491-1495. doi: 10.1007/s11250-016-1088-4

De Oliveira, A. R. A., Pereira, M. L. A., de Jesus Pereira, T. C., de Oliveira Silva, H. G., da Silva, E. R., de Carvalho Dutra, I., Correia, G. S., Dos Santos Soares, V. P., De Oliveira Ribas, K. P., and Porto, M. R. (2021). Enriched Mesquite Piperidine Alkaloid Extract Improves the Performance in Growing Goats. International Journal of Environmental & Agriculture Research: 7, 77-90. doi: 10.5281/zenodo.4647052

- Magalhães, T. S., Carvalho, G. G. P., Santos, E. M., Júnior, J. F., Pina, D. S., Pinto, L. F. B., and Leite, L. C. (2019). Effect of cottonseed processing and chitosan supplementation on lamb performance, digestibility and nitrogen digestion. J. Agric. Sci. 157, 636-642. doi: 10.1017/S0021859619000911
- Nicory, I. M. C., de Carvalho, G. G. P., Ribeiro, O. L., Santos, S. A., da Silva, F. F., Silva, R. R., and Freitas Jr, J. E. (2015). Productive and metabolic parameters in lambs fed diets with castor seed meal. Livest. Sci. 181, 171-178. doi: 10.1016/j.livsci.2015.09.015

*****Oliveira, C. R. N., Santos, S. A., Mariz, L. D. S., Carvalho, G. G. P., de Azevêdo, J. A. G., Tosto, M. S. L., and Santos, A. C. S. (2020). Dietary phase-feeding as feedlot strategy for Santa Ines lambs: performance, N retention and meat quality. Livest. Sci. 239, 104-106. doi: 10.1016/j.livsci.2020.104106 ****This manuscript contain two experiments, than it must be accounted for 2 studies in our dataset.***

Pereira, E. S., Campos, A. C. N., Heinzen, E. L., Barbosa Filho, J. A. D., Carneiro, M. S. S., Fernandes, D. R., Bezerra, L. R., Oliveira, R. L. (2018). Effect of dietary reduction and sex class on nutrient digestibility, nitrogen balance, excreted purine derivatives and infrared thermography of hair lambs. J. Agric. Sci. 156, 1028-1038. doi: 10.1017/S0021859618001016

Pereira, E. S., Pereira, M. W. F., Arruda, P. C. L., Cabral, L. S., Oliveira, R. L., Mizubuti, I. Y., and Carneiro, M. S. S. (2016). Effects of different lipid sources on intake, digestibility and purine derivatives in hair lambs. J. Anim. Physiol. Anim. Nutr. 100, 723-730. doi: doi.org/10.1111/jpn.12438

Pereira, G. A., Santos, E. M., Araújo, G. G. L., Oliveira, J. S., Pinho, R. M. A., Zanine, A. D. M., and Nascimento, T. V. C. (2019). Isolation and identification of lactic acid bacteria in fresh plants and in silage from Opuntia and their effects on the fermentation and aerobic stability of silage. J. Agric. Sci. 157, 684-692. doi: 10.1017/S0021859620000143

Pereira, T. C. D. J., Pereira, M. L. A., Almeida, P. J. P., Pereira, C. A. R., Santos, A. B. D., and Santos, E. D. J. D. (2013). Mesquite pod meal in diets for Santa Inês sheep: ingestive behavior. Acta Scientiarum. Animal Sciences. 35, 201-206. doi: 10.4025/actascianimsci.v35i2.16221

Pereira, M. L. A., Pereira, T. C. J., Silva, H. G. O., Cruz, J. F., Almeida, P. J. P., Santos, A. B., Santos, E. J., and Peixoto, C. A. M. (2013). Substitution of corn by mesquite pod meal in pellet diets for lambs: nitrogen compounds metabolism. In: Energy and protein metabolism and nutrition in sustainable animal production. Oltjen, J. W., Kebreab, E., and Lapierre, H. (Eds.). Wageningen Academic Publishers, Wageningen, pp. 93-94.

Pinho, R. M. A., Santos, E. M., de Oliveira, J. S., de Carvalho, G. G. P., da Silva, T. C., Macêdo, A. J. D. S., and Zanine, A. D. M. (2018). Does the level of forage neutral detergent fiber affect the ruminal fermentation, digestibility and feeding behavior of goats fed cactus pear?. Anim. Sci. J. 89, 1424-1431. doi: 10.1111/asj.13043

Santos, A. B., Pereira, M. L. A., Silva, H. G. O., Pedreira, M. S., Carvalho, G. G. P., Ribeiro, L. S. O., and Moreira, J. V. (2014). Nitrogen metabolism in lactating goats fed with diets containing different protein sources. Asian. Australas. J. Anim. Sci. *27*, 658. doi: 10.5713/ajas.2013.13493

Santos, A. C. S., Santos, S. A., Carvalho, G. G. P., Mariz, L. D. S., Tosto, M. S. L., Valadares Filho, S. C., and Azevedo, J. A. G. (2018). A comparative study on the excretion of urinary metabolites in goats and sheep to evaluate spot sampling applied to protein nutrition trials. J. Anim. Sci. *96*, 3381-3397. doi: 10.1093/jas/sky198

Silva, L. O., Carvalho, G. G. P., Tosto, M. S. L., Lima, V. G. O., Cirne, L. G. A., Santos Pina, D., and Azevedo, J. A. G. (2020). Digestibility, nitrogen metabolism, ingestive behavior and performance of feedlot goats fed high-concentrate diets with palm kernel cake. Livest. Sci. 241, 104226. doi: 10.1016/j.livsci.2020.104226

- Silva, R. V. M. M., de Carvalho, G. G. P., Pires, A. J. V., Pereira, M. L. A., Pereira, L., Campos, F. S., and Almeida Rufino, L. M. (2016). Nitrogen balance, microbial protein synthesis and ingestive behavior of lambs fed diets containing cottonseed cake in substitution of soybean meal. Semina: Ciências Agrárias. 37, 2155-2166. doi: 10.5433/1679-0359.2016v37n4p2155
